# Transplantation of induced mesenchymal stem cells for treating chronic renal insufficiency

**DOI:** 10.1371/journal.pone.0176273

**Published:** 2017-04-26

**Authors:** Xing-hua Pan, Jing Zhou, Xiang Yao, Jun Shu, Ju-fen Liu, Jian-yong Yang, Rong-qing Pang, Guang-ping Ruan

**Affiliations:** 1 The Cell Biological Therapy Center, Kunming General Hospital of People’s Liberation Army, Kunming, Yunnan Province, China; 2 Stem Cells and Immune Cells Biomedical Techniques Integrated Engineering Laboratory of State and Regions, Kunming, Yunnan Province, China; 3 Cell Therapy Technology Transfer Medical Key Laboratory of Yunnan Province, Kunming, Yunnan Province, China; Universita degli Studi di Torino, ITALY

## Abstract

Discovering a new cell transplantation approach for treating chronic renal insufficiency is a goal of many nephrologists. In vitro-cultured peripheral blood mononuclear cells (PBMCs) were reprogrammed into induced mesenchymal stem cells (iMSCs) by using natural inducing agents made in our laboratory. The stem cell phenotype of the iMSCs was then identified. Unilateral ureteral obstruction (UUO) was used to create an animal model of chronic renal insufficiency characterized by renal interstitial fibrosis. The induced and non-induced PBMCs were transplanted, and the efficacy of iMSCs in treating chronic renal insufficiency was evaluated using a variety of methods. The ultimate goal was to explore the effects of iMSC transplantation on the treatment of chronic renal insufficiency, with the aim of providing a new therapeutic modality for this disease.

## Introduction

Chronic kidney disease is one of the leading health problems worldwide, and the incidence of this disease is increasing every year [1]. Common treatments for chronic kidney disease, such as hemodialysis and peritoneal dialysis, can neither fundamentally improve renal pathological damage nor effectively prevent the occurrence of various complications. Renal transplantation can solve the problem, but the lack of donor organs and immune rejection following transplantation limit the widespread application of this treatment method. Most patients lose opportunities while waiting for renal transplantation. Therefore, the search for effective treatments remains a key issue in treating kidney disease. Stem cell transplantation may provide effective treatment for kidney disease. Stem or progenitor cell therapies offer an alternative strategy for modulating complex disease processes by suppressing multiple pathogenic pathways and promoting pro-regenerative mechanisms. Mesenchymal stem cells (MSCs) have shown particular promise in this regard based on their availability from adult tissues and their diverse mechanisms of action, including secretion of paracrine anti-inflammatory and cytoprotective factors [2].

The unilateral ureteral obstruction (UUO) model was implemented to study the current technology used in the prevention and control of kidney disease. The model is characterized by significant glomerular sclerosis and renal interstitial fibrosis. Reduced renal mass leads to compensatory hypertrophy of the kidney, an increased filtration rate, glomerular and systemic hypertension, and, finally, the formation of glomerular sclerosis. Studies have found that transforming growth factor (TGF)-β1, angiotensin II, tumor necrosis factor (TNF)-α, osteopontin (OPN) and collagen I, III, and IV are all elevated [3]. The cytokine TGF-β1 strongly induces fibrosis, which can lead to glomerulosclerosis and renal interstitial fibrosis [4].

At present, many studies suggest that bone marrow mesenchymal stem cells (BMSCs) can reduce renal fibrosis by regulating immune function and tissue remodeling. Qian et al. [5] reported that injured kidney tissue induced rat and human MSCs to differentiate into renal tubular epithelial-like cells in vitro and in vivo and that exogenous human MSCs could home specifically to injured regions and efficiently cure rat acute renal failure (ARF). These results demonstrate that cell therapy has potential as a novel intervention in ARF. Some studies have explored whether these effects can help repair the peritubular capillary plexus and improve the status of tubular and intercellular hypoxia. These studies found that homologous BMSCs can stimulate endothelial cells to repair the peritubular capillaries, thereby improving the status of renal tubule and intercellular hypoxia [6, 7]. Togel et al. reported that vascular endothelial growth factor (VEGF) is an important factor produced by BMSCs in the kidney [8]. In recent years, there have been reports that endothelial progenitor cells also have beneficial effects on chronic kidney disease [9]. Numerous studies have demonstrated that using BMSCs in treating chronic renal fibrosis has a beneficial effect [10–15]. In recent years, the possible roles of other cellular therapies (i.e., microvesicles/exosomes) have been discussed. Conforti et al. [16] reported that compared to their cellular counterparts, microvesicles showed weaker in vitro immunomodulatory effects on T-cell proliferation and antibody formation. Yu et al. [17] reported that exosomes play an important role in intercellular signaling and exert a regulatory function by transporting bioactive molecules. In particular, exosomes have been identified as a type of cardioprotective component in MSC secretion of paracrine factors and have been demonstrated to reduce myocardial injury. The advantages of MSCs are that they are multipotent cells able to differentiate into various mature cell lineages and that they show immunomodulatory effects by inhibiting T-cell proliferation. However, a major disadvantage of using MSCs is their likelihood of inducing malignant transformation. MSCs are undifferentiated cells that possess immunomodulatory and tissue trophic properties as well as the ability to differentiate into multiple cell types. Studies in animal models of chronic renal failure have revealed a unique potential of these cells for regenerating the damaged kidney and improving its function [18].

The use of induced pluripotent stem cells (iPSCs) is a recent advance in stem cell research. By the end of 2007, Yamanaka’s [19] team used a lentiviral vector to introduce four transcription factor genes, Oct4, Sox2, c-Myc, and Klf4, into fetal, adult, and aging somatic cells. These transcription factors were successful in reprogramming fibroblasts into embryonic stem (ES) cells. The use of iPSCs appears promising for kidney repair and regeneration. Using various emerging differentiation protocols, human iPSCs can be derived from somatic cells, and iPSCs can be converted into self-organizing kidney organoids. Several groups have successfully generated kidney organoids that produce urine upon transplantation into a mouse host. Additional advances in culturing nephron progenitors in vitro may provide another source for kidney engineering [20].

In this study, we utilized inducing agents that had previously been developed in our laboratory [21]. Adult peripheral blood mononuclear cells (PBMCs) were harvested, separated, and purified; finally, the inducing agents were added to the culture, reprogramming the PBMCs into induced mesenchymal stem cells (iMSCs). Surface markers for stem cells were used to identify iMSCs at the molecular level. At the same time, the therapeutic effects of using iMSCs were comprehensively evaluated. This study used PBMCs derived from peripheral blood because they can be harvested more conveniently than skin- or bone marrow-derived cells. The separation conditions were relatively stable. This study used the novel induction method of treatment with inducing agents derived from animal oocyte extracts [21] to reprogram PBMCs into iMSCs. This technique has several advantages: the risks involved with gene transfection can be avoided, the induction efficiency can be improved, and the experiments are easier and less expensive than with other methods. The main disadvantage of this technique is that the function of the induced PBMCs remains unclear and thus requires further study. The use of iMSCs may hold much promise in kidney repair and regeneration and may prove to be comparable to iPSCs as a source of stem cells for use in these therapies.

## Materials and methods

### 1. Establishment of experimental groups of a rabbit model of chronic renal failure

Forty wild-type Japanese white rabbits were numbered according to body mass and randomly divided into the following two groups: a normal control group (n = 10) of healthy rabbits not undergoing any treatment and the unilateral ureteral obstruction (UUO) model group (n = 30) of rabbits undergoing left ureteral ligation.

The rabbits were anesthetized with 3% sodium pentobarbital at a dose of 1 ml/kg injected intravenously into the ear vein. Hair was removed from the surgical field, the rabbit was placed supine on a sterile operating plate, and routine disinfection with sterile towels was conducted. A longitudinal incision was made near the top of the left kidney along the midline, and the incision was opened layer by layer to a depth of approximately 3–5 cm. The left kidney and ureter were isolated, and while gently holding the middle portion of the ureter with tissue forceps, a 2 cm length of the ureter was isolated and ligated with a 5–0 surgical suture. The kidney capsule and surrounding tissue were protected, and the muscle and skin were sutured. Penicillin was injected intramuscularly for 3 days to prevent infection of the wound.

The UUO group was divided into the following three subgroups: a non-induced group (n = 10) of UUO animals transplanted with non-induced PBMCs, an induced group (n = 10) of UUO animals transplanted with induced PBMCs, and a UUO control group (n = 10) of UUO animals that did not undergo any transplantation. The effects of treatment with induced PBMCs (iMSCs) were comprehensively evaluated through several assays. All experimental protocols were approved by the Experimental Animal Ethics Committee of Kunming General Hospital of People’s Liberation Army.

### 2. In vitro cultivation, induction and identification of autologous PBMCs

Density gradient centrifugation with stratified Ficoll-Hypaque was used to isolate the mononuclear cells from the peripheral blood. Inducing agents derived from chicken egg oocytes [21] were used to reprogram the PBMCs into iMSCs.

Quantitative PCR methods were used to detect the relative levels of gene expression of OCT4 and NANOG in the induced cells and the non-induced cells. Following induction, the PBMCs with or without exposure to the inducing agents were cultured for three days and collected for RNA extraction. Following reverse transcription, quantitative PCR was performed to quantify the relative levels of OCT4 and NANOG gene expression, using GAPDH as an internal control.

Flow cytometry was utilized to detect OCT4 and NANOG protein expression in the non-induced and induced PBMCs. Again, following the addition of the inducing agents, both the induced and non-induced PBMCs were cultured for three days, collected, and centrifuged. The supernatant was discarded, and the cell pellets were resuspended in 200 μl of 4% paraformaldehyde in PBS and fixed for 10 min at room temperature. Then, 1 ml of Perm/Wash Buffer was added, the solution was centrifuged, and the supernatant was discarded. The precipitate was resuspended in 50 μl of Perm/Wash Buffer. Then, 1.5 μl of OCT4-PE (eBioscience) and Rat IgG2a *k* Isotype Control PE were added for labeling. 20 μl of NANOG-PE (BD Biosciences) and PE Mouse IgG1, *k* Isotype Control were added for labeling, and the samples were incubated at room temperature for 1 h in the dark. Finally, the cells were washed with Perm/Wash Buffer, and the precipitate was resuspended in 400 μl of PBS. The samples were then analyzed by flow cytometry.

### 3. Transplantation and efficacy evaluation of autologous iMSCs

The establishment of an animal model of chronic renal insufficiency was evaluated by comparing the normal control group, the UUO control group, the non-induced UUO group and the induced UUO group. The efficacy of using iMSCs for treating kidney disease was evaluated by comparing the induced and non-induced groups.

Beginning two weeks after UUO, the induced and non-induced PBMCs were transfused into the animals via the ear vein once per week for four weeks. This protocol was based on human stem cell treatments: humans usually receive stem cell treatments once per week for four weeks.

Serum creatinine (Scr) and blood urea nitrogen (BUN) contents were measured in peripheral blood samples collected via the ear vein for each group, and the values were compared between groups.

SPECT was used to monitor the glomerular filtration rate (GFR) and renal blood flow. The relative content of TGF-β1 in the renal tissues was measured by semi-quantitative PCR, and TGF-β1 expression was analyzed by immunohistochemistry. Ultrathin renal tissue sections were prepared, and the internal cellular ultrastructure was observed by SEM.

To perform semi-quantitative PCR to quantify the expression of TGF-β1, total RNA was extracted from the kidney tissues, the gray value of the sample was measured, and the relative expression levels of TGF-β1 were calculated according to the following formula: Relative expression = sample gray value/internal control gray value.

The primer sequences for TGF-β1 and the internal control gene (18S rRNA) were obtained from gene sequences in GenBank by using the primer design software Primer Premier 5.0. The primer sequences for the target genes were also identified from the literature [22]. All primers were synthesized by Bioengineering Co., Ltd. (Shanghai), and the primer sequences are shown in Table 1.

**Table 1:** Primer sequences for the target and control genes (18S rRNA).

Gene	Primer sequences	Accession No.
18S rRNA	F: 5’-GCG GCT TTG GTG ACT CTA-3’	BC063166
	R: 5’-CTG CCT CCT TGG ATG TG-3’	
TGF-β1	F: 5’-TAA TGG TGG ACC GCA ACA ACG-3’	A23751
	R: 5’-CTT GCT GTA CTG TGT GTC CAG-3’	

SPECT monitoring was performed in randomly selected rabbits from each group using radionuclide dynamic renal scanning (Discovery VH dual-head coincidence SPECT, General Electric, USA). The Department of Nuclear Medicine at Kunming General Hospital assisted with the imaging.

To perform SEM, the kidney tissues were rinsed in electron microscopy rinsing solution, fixed for 2 h with 1% osmic acid, and dehydrated using an alcohol series. The samples were then incubated at 4°C overnight in saturated uranyl acetate electron staining solution, embedded in epoxy resin, and allowed to polymerize at 68°C for two days. Finally, the tissues were thinly sliced, placed on a copper grid, and stained with lead. A CM-120 electron microscope was used to observe the samples.

### 4. Statistical analyses

The experimental data are presented as the means ± standard deviation. Using the statistical software package SPSS 17.0, pairwise differences between the groups were compared using the independent-samples t-test and the paired t-test, and values of P≤0.05 were considered significant.

## Results

### 1. Cell culture and induction

#### 1.1 Isolation and culture of PBMCs

**Fig 1:**
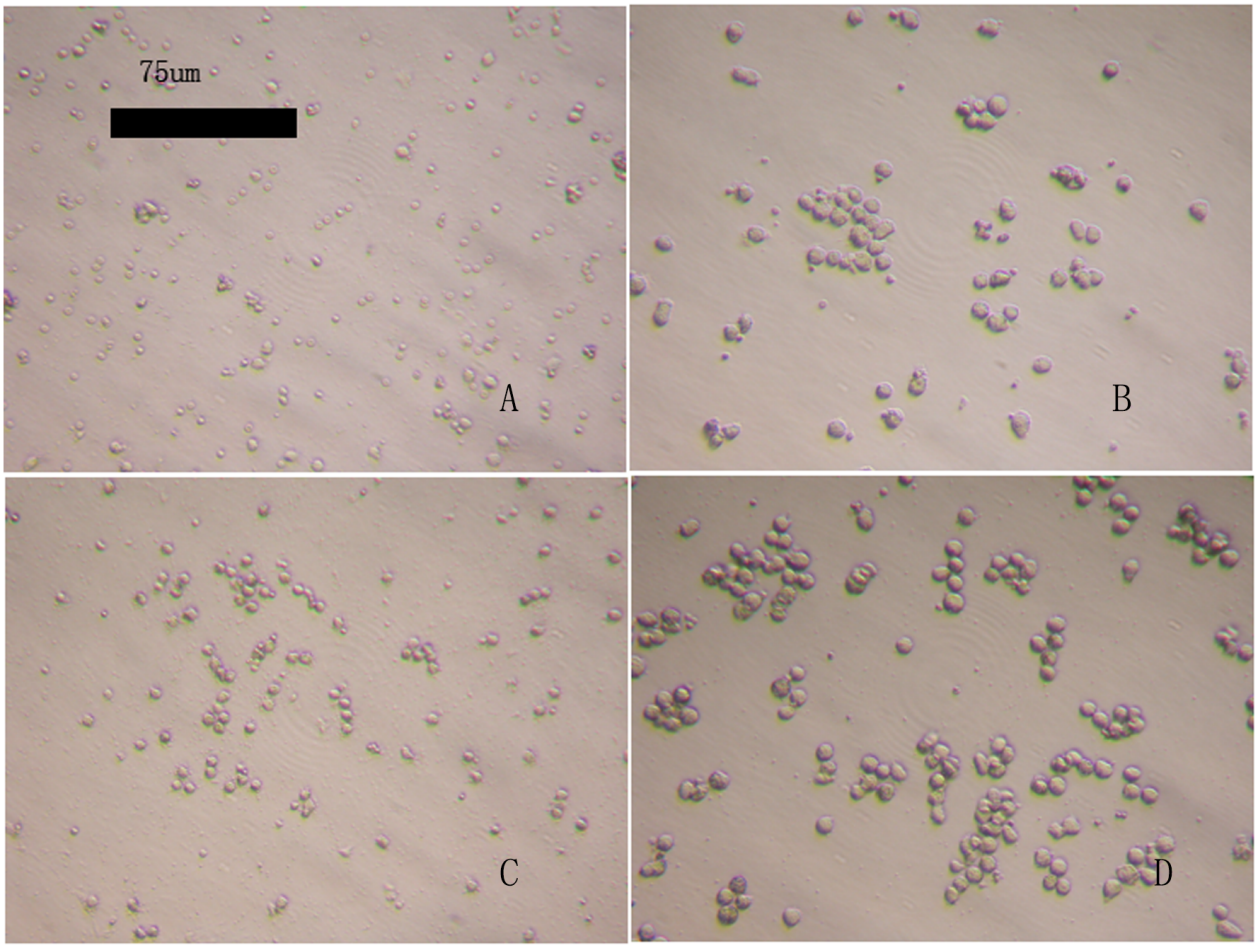
Peripheral blood mononuclear cells (PBMCs). A, B: Non-induced PBMCs at 0 h and 72 h, respectively. C, D: Induced PBMCs at 0 h and 72 h, respectively. Fig 1D shows that induced PBMCs cultured for 72 h have grown into colonies with higher numbers and larger sizes of cells.

Fig 1A shows freshly isolated and purified PBMCs, which have an ovoid and slightly flat morphology. Fig 1B shows PBMCs on the third day of primary culture; the number of cells was significantly reduced, and the cells were larger.

#### 1.2 Induced culture of PBMCs

Fig 1C shows that the freshly isolated and purified PBMCs were oval in shape and slightly flat. Fig 1D shows the induced PBMCs after 72 h in culture, at which point the cells had grown into colonies and showed higher numbers and larger sizes of cells.

#### 1.3 Identification of the induced PBMCs

**Fig 2:**
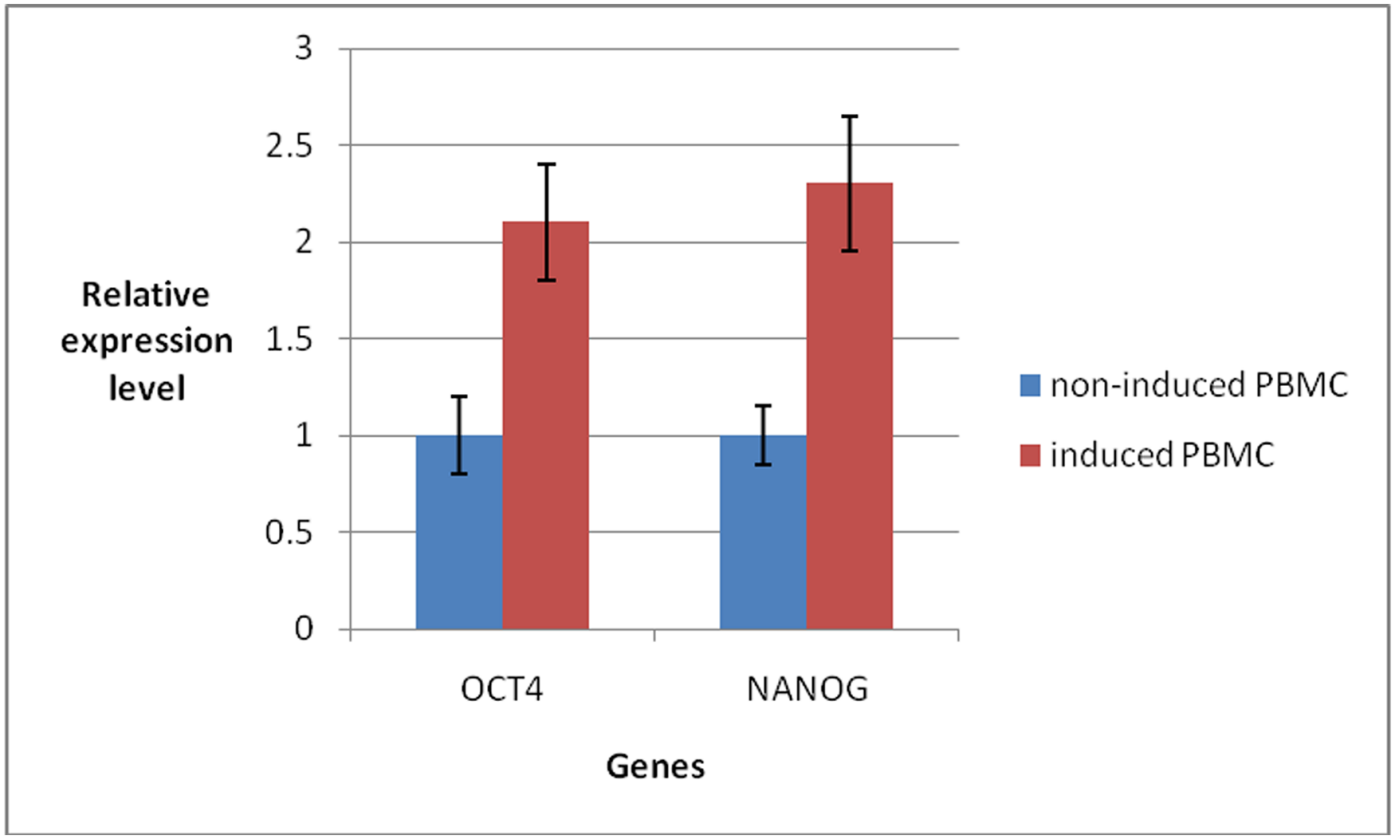
Evaluation of relative OCT4 and NANOG gene expression via quantitative PCR. The relative expression levels of OCT4 and NANOG increased significantly in the induced PBMCs.

**NANOG and OCT4 gene expression.** Using quantitative PCR with the relative gene expression levels of OCT4 and NANOG normalized to the levels in the non-induced PBMCs, the relative expression levels of OCT4 and NANOG in the induced PBMCs at 3 days after induction were 2.1 and 2.3, respectively (Fig 2). These increases in the relative expression levels of OCT4 and NANOG in the induced PBMCs were significant.

**Fig 3:**
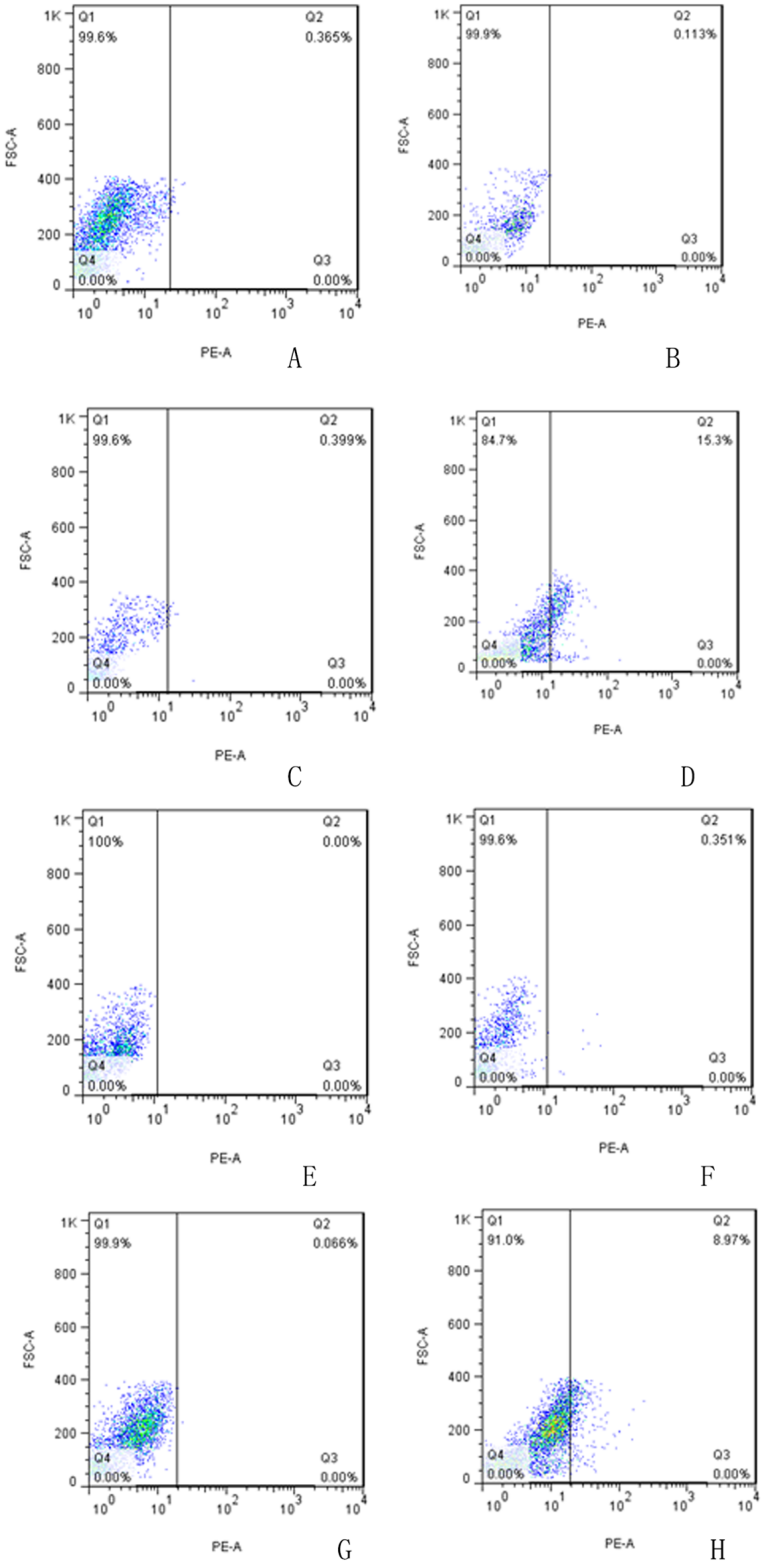
Flow cytometric detection of OCT4 and NANOG protein expression in the non-induced and induced PBMCs. A, B, C, D: OCT4 protein expression. E, F, G, H: NANOG protein expression. A, B, E, F: Non-induced PBMCs. C, D, G, H: Induced PBMCs. A, C, E, G: Isotype Control. After induction, the rates of OCT4 and NANOG protein expression were significantly increased.

**Flow cytometric detection of OCT4 and NANOG protein expression.** The proportions of the non-induced PBMCs expressing OCT4 and NANOG were 0.113 and 0.351%, respectively. After 3 days, the proportions of induced PBMCs expressing OCT4 and NANOG were 15.3 and 8.97%, respectively (Fig 3). After induction, the rates of OCT4 and NANOG protein expression were significantly increased.

### 2. Serum creatinine and blood urea nitrogen levels

The BUN and Scr contents after 4 weeks of cell therapy are shown in Table 2. The BUN and Scr values in the UUO control group were significantly higher than those in the normal controls. After 4 weeks of treatment, the BUN and Scr values in the non-induced group were similar to those in the UUO control group; however, the BUN and Scr values in the induced group were significantly lower than those in the UUO control group.

**Table 2:** Changes in the BUN and Scr contents in each group four weeks after treatment (means ± SD, n = 10).

	Number	Blood urea nitrogen (mmol/L)	Serum creatinine (μmol/L)
**Normal group**	10	3.34±2.35	105.32±10.64
**UUO control group**	10	10.81±1.33	155.83±12.32
**Non-induced group**	10	11.18±1.34	153.5±10.48
**Induced group**	10	6.95±1.61*	113.1±14.19*

*P<0.05 compared with the other groups.

### 3. Comparison of the glomerular filtration rate (GFR) and renal blood flow for each group

**Fig 4:**
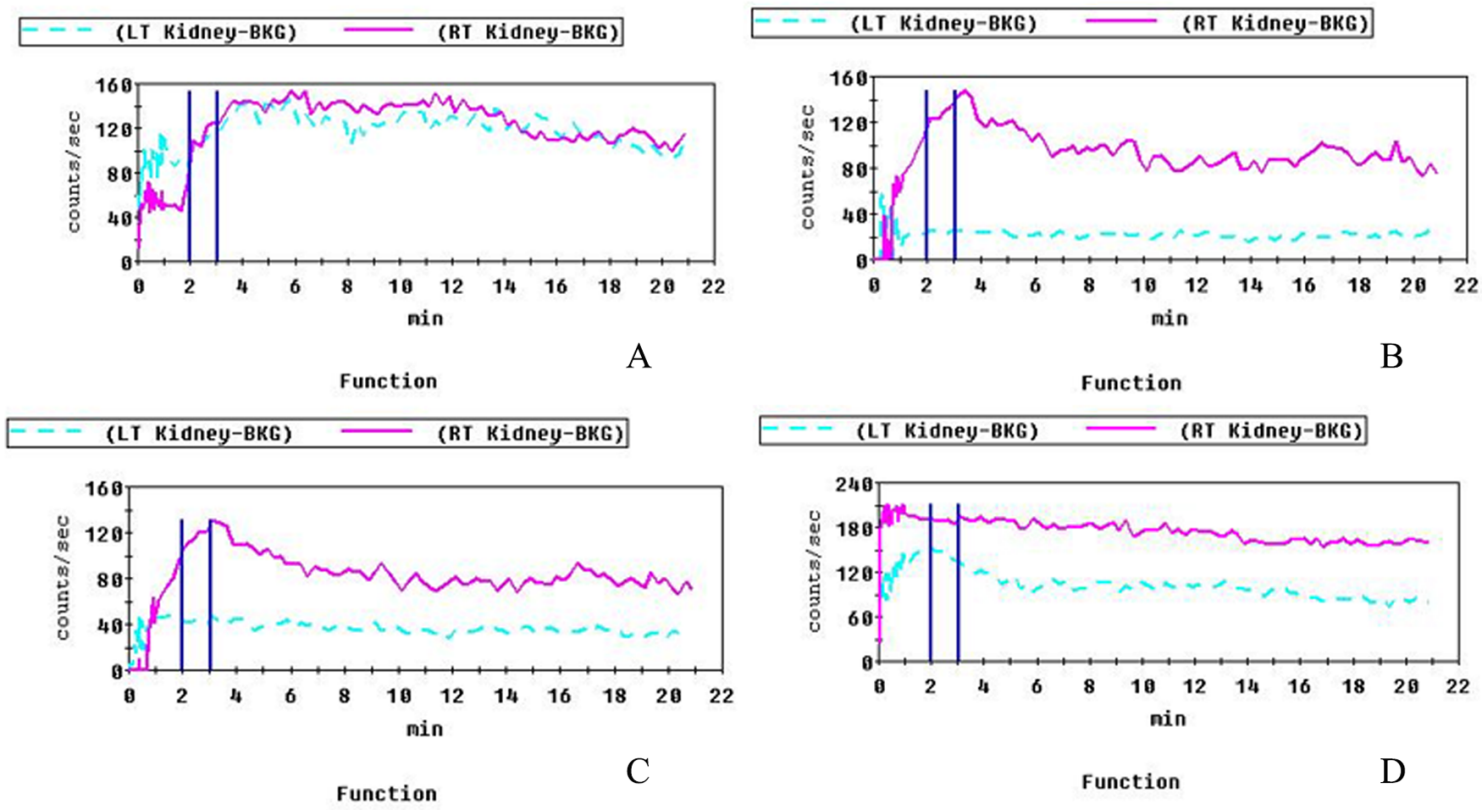
Changes in kidney function. A: Normal control group: GFR is 31.1; B: UUO control group: GFR decreases to 8.6; C: non-induced group: GFR is 15.0; D: induced group: GFR returns to 30.9. The renal blood flow increased significantly in the induced group at 4 weeks.

The SPECT test results showed that after the fourth week after cell transplantation, the GFR of the normal group was 31.1. The renal blood flow was decreased in the UUO group, indicating that the ligated side had reduced or even complete loss of kidney function; the GFR value was reduced to 8.6. After treatment, the GFR value in the non-induced group was 15.0, and the renal blood flow remained low. The GFR value returned to 30.9, and the renal blood flow increased significantly in the induced group at 4 weeks. These results are shown in Fig 4.

### 4. Immunohistochemical results for TGF-β1 (P<0.05)

**Fig 5:**
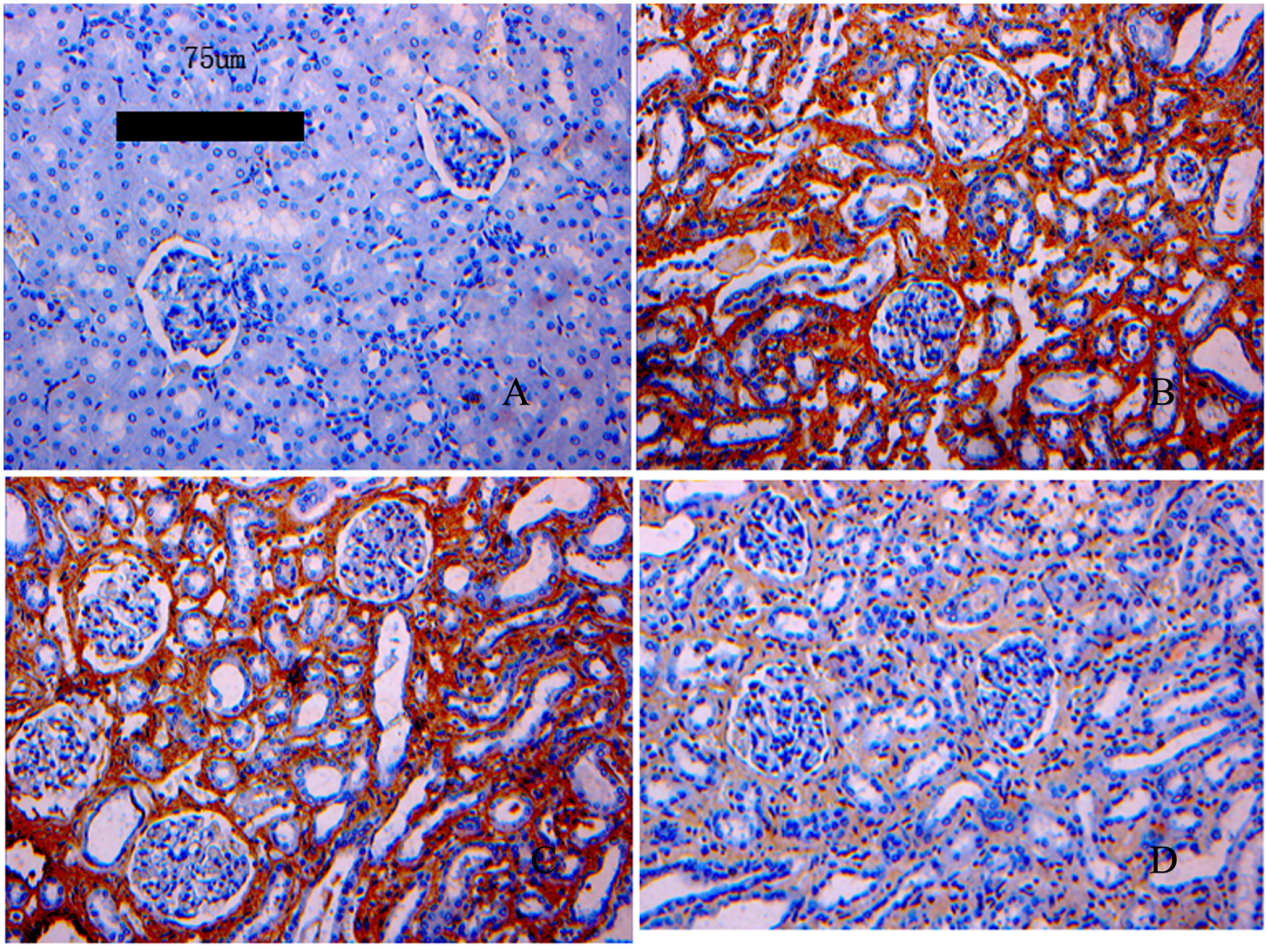
Expression of TGF-β1 as shown by immunohistochemistry. A: Normal control group; B: UUO control group; C: non-induced group; D: induced group. The expression of TGF-β1 was lower in the induced group than in the non-induced and UUO control groups.

Using Image-Pro Plus multimedia color pathological image analysis software, the expression of TGF-β1 was observed to be much lower in the normal control group than in the UUO control group. The expression of TGF-β1 was lower in the induced group than in the non-induced group. The results are shown in Table 3 and Fig 5.

**Table 3:** Immunohistochemical results for TGF-β1 expression in rabbit kidney (means ± SD, n = 10).

	Number	Immunohistochemistry
**Normal group**	10	1.405±0.91
**UUO control group**	10	27.15±4.13
**Non-induced group**	10	25.50±4.14
**Induced group**	10	19.46±1.89*

*P<0.01 compared with other groups.

### 5. Semi-quantitative PCR results for TGF-β1 expression

**Fig 6:**
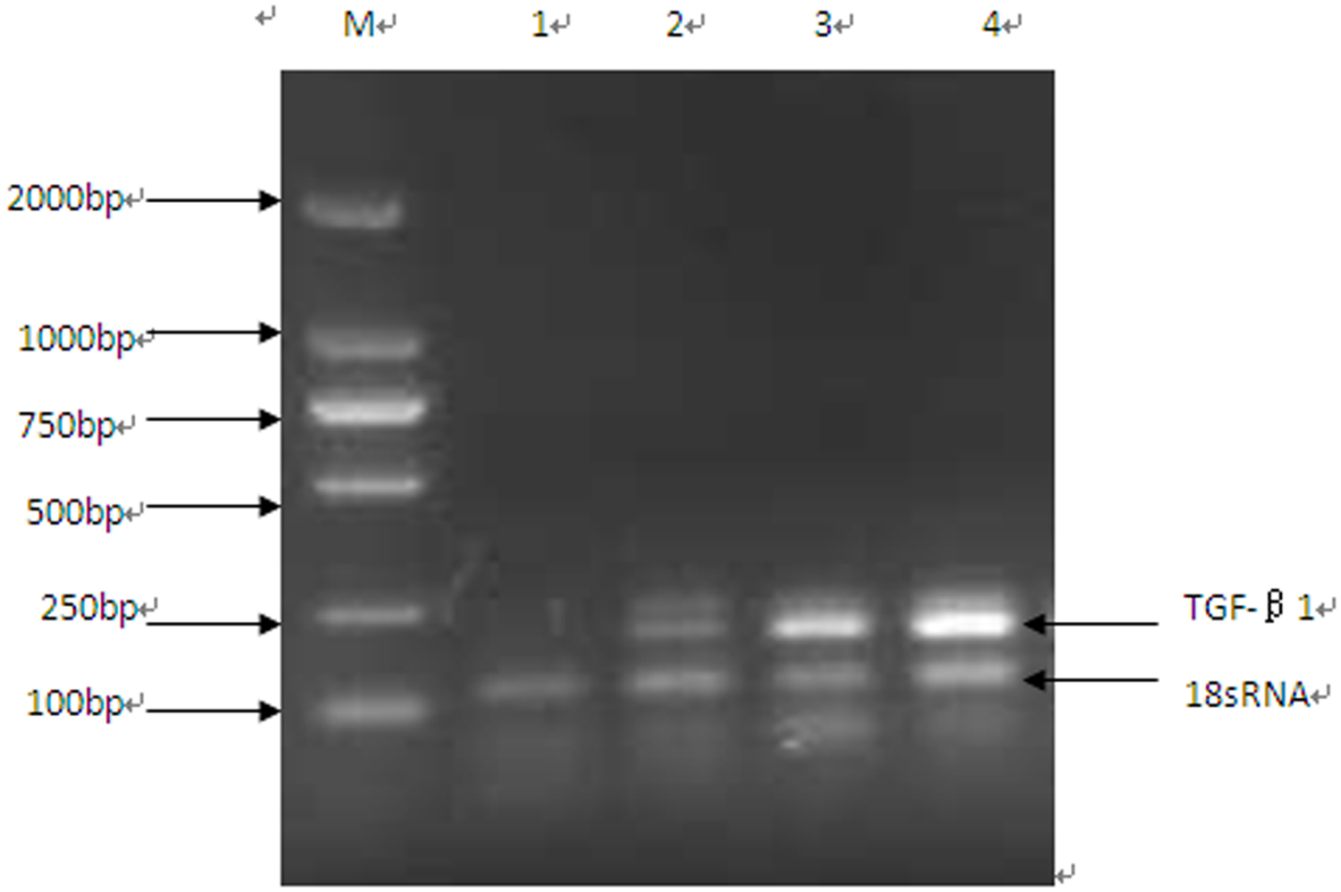
Semi-quantitative PCR detection of TGF-β1 expression. M, Marker; 1, normal control group; 2, induced group; 3, non-induced group; 4, UUO control group. TGF-β1 expression was significantly lower in the induced group than in the UUO control group and the non-induced group.

The lane labeled M is the marker, and the first lane shows the normal control group with no expression of TGF-β1. The second lane shows the induced group, with the first band indicating TGF-β1 expression and the second band indicating the control gene, 18S rRNA. The third lane shows the non-induced group. Finally, the fourth lane shows the UUO control group, with the first band indicating clear expression of TGF-β1 and the second band showing the 18S rRNA control gene. The results are shown in Table 4 and Fig 6. TGF-β1 expression was significantly lower in the induced group than in the UUO control group and the non-induced group.

**Table 4:** Semi-quantitative PCR results for the relative expression of TGF-β1 (means ± SD, n = 5).

	Internal reference	Sample	Relative expression
**Normal group**	703.21±5.6	0±0	0±0
**UUO control group**	1297.42±8.9	2392.87±9.2	1.844±0.56
**Non-induced group**	1513.55±9.8	2416.49±9.9	1.596±0.65
**Induced group**	1155.5±9.7	1605.5±8.9	1.389±0.54*

*P<0.01 compared with other groups.

### 6. SEM observation of renal tissue fibrosis in each group

**Fig 7:**
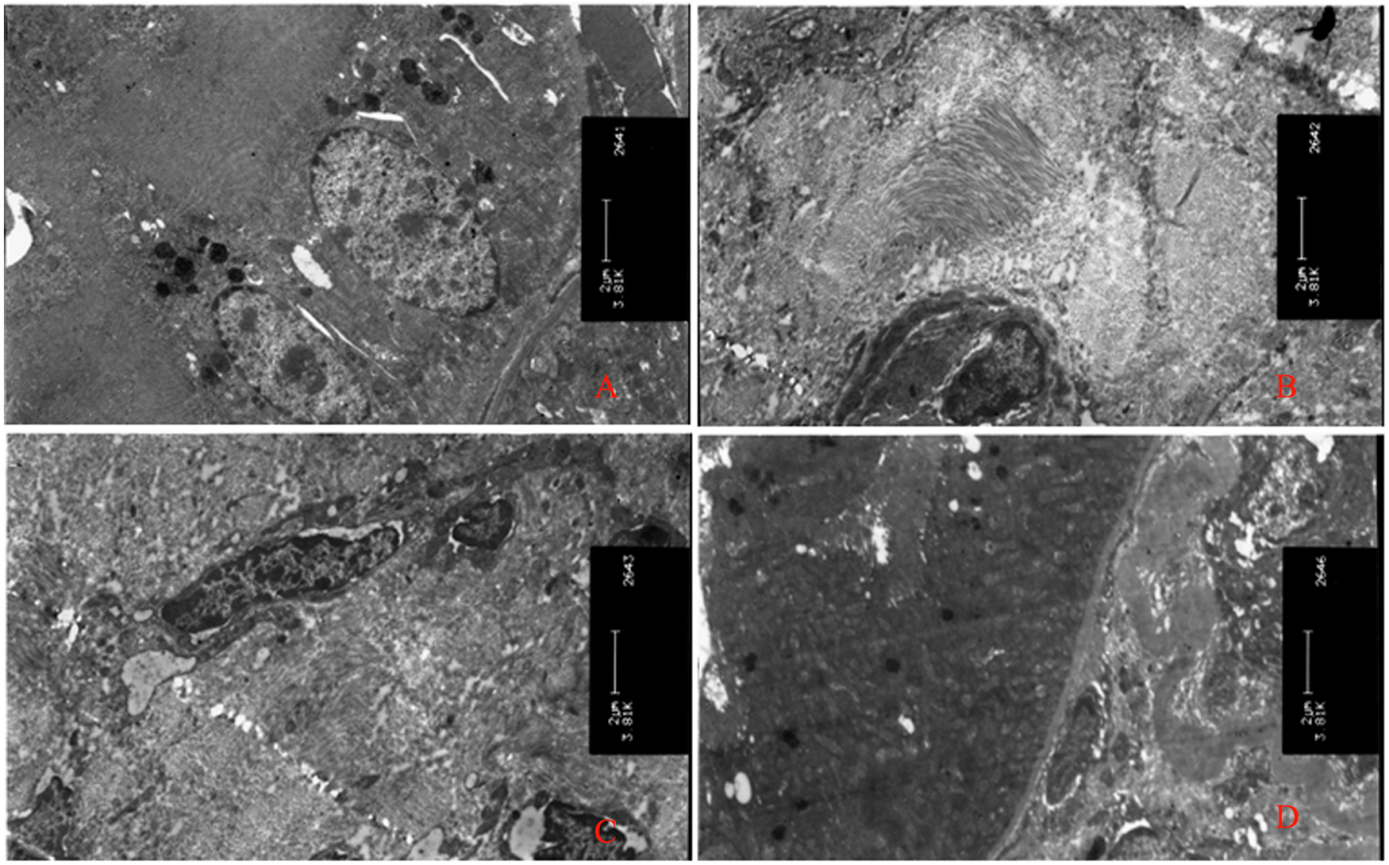
Kidney tissue SEM results (3.81 × 1000 times). A. Normal group: normal renal tubular epithelial cells; B. UUO control group: extensive renal interstitial fibrosis with fibroblast proliferation; C. non-induced group: renal interstitial fibrosis remains widespread; D. induced groups: no obvious renal interstitial fibrosis and visible normal tubular and tubular epithelial cells.

The normal control group showed normal renal tubular epithelial cells. The UUO control group exhibited renal interstitial fibrosis with extensive proliferation of fibroblasts. The non-induced group showed widespread renal interstitial fibrosis. Finally, in the induced group, there was no obvious renal interstitial fibrosis, and normal tubular and tubular epithelial cells were visible, as shown in Fig 7.

## Discussion

Research on the use of stem cells for kidney disease is currently primarily focused on adult stem cells. In particular, bone marrow stem cells are being studied for use in therapy for chronic renal failure; however, their proliferation, differentiation, and plasticity are not as robust as those of embryonic stem (ES) cells. To complicate matters further, there are ethical and safety issues regarding the use of ES cells. Induced pluripotent stem cells (iPSCs) are emerging as a new source of stem cells, and they share certain biological properties with ES cells. If it is possible to overcome the technical challenges associated with promoting the differentiation of iPSCs and their development into healthy kidney cells, these cells will provide hope for kidney regeneration.

Currently, iPSCs can be reprogrammed by the following methods: 1) cloning technology, wherein somatic cell nuclei are transferred into enucleated oocytes and incubated to obtain ES cells; 2) use of early embryo extracts in conjunction with other special factors to reprogram mature cells into stem cells; and 3) transfection of specific transcription factors into somatic cells to directly convert them into stem cells. Several cell types can be identified in the adherent fraction of bone marrow mononuclear cells, including more primitive and embryonic-like stem cells, mesenchymal stem cells (MSCs), lineage-committed progenitors, and mature cells such as osteoblasts and fibroblasts. The accumulation of a self-renewing MSC-like subpopulation with low expression levels of aging markers provides a valuable tool for regenerative medicine [23]. Over the last few years, many papers have been published by research groups around the world investigating the clinical application of stem cells for renal failure; most of this work has used alternative sources of MSCs or sub-fractions of stem cells, which are not only useful for kidney regeneration but also more applicable for Good Manufacturing Practice (GMP) production, thereby avoiding the involvement of gene therapy, animal-derived supplements or other additives. However, in this paper, we present a novel reprogramming strategy using a new cell type (iMSCs) for the treatment of chronic renal insufficiency in rabbits and investigate the efficacy and safety of this new cell type.

In this study, a laboratory-made oocyte suspension [21] was used to induce rabbit PBMCs according to previous protocols from our laboratory. Expression of OCT4 and NANOG at the gene and protein levels is only observed in stem cells, and these transcription factors are markers of stem cell pluripotency. Quantitative PCR and flow cytometry identified the stem cell markers OCT4 and NANOG in the induced PBMCs. After induction, the expression levels of both markers were elevated. In this method, unlike gene transfection with synthetic chemicals, there was no damage to the cells, the culture conditions were simple and non-toxic, the induction efficiency was high, and there was no risk for gene transfer. The present study provides a new method for inducing somatic cells to develop into iMSCs.

In this study, the UUO technique was used as a model of chronic renal insufficiency and renal interstitial fibrosis. Compared with other physical methods, it is a relatively simple surgical procedure, with a small incision, a low chance of infection, and high rate of animal survival. After ureteral obstruction, the serum creatinine and urea nitrogen levels were significantly higher in the first eight weeks. As determined by immunohistochemistry, after 8 weeks, there was tubular dilation or atrophy, significant interstitial fibrosis, significantly increased TGF-β1 expression, and apparent glomerular sclerosis in the UUO group. Using SPECT, it was easy to see that the GFR in the first eight weeks was significantly lower than that in the normal group, reaching conditions of chronic renal insufficiency.

This experiment studied the use of iMSCs as a potential treatment for renal fibrosis. Based on biochemical indexes, functional assays, histological evaluation, and molecular marker characterization, stem cell therapy appears to ameliorate fibrosis and inflammation. Therefore, we believe that we have found a new method for treating chronic kidney disease. Using a chronic renal injury model, injection of induced PBMCs once per week for four weeks was able to significantly improve blood biochemical parameters such as Scr and BUN levels, which dropped significantly. Four weeks after the induced PBMC therapy, the GFR was significantly improved, as were blood flow and other measures of kidney function. To further characterize the improvement in renal function following the treatment with induced PBMCs, the kidney structure was semi-quantitatively analyzed, confirming a reduction in the degree of renal fibrosis. Because induced PBMCs are multipotent cells, they may prevent kidney damage by differentiating into renal cells and participating in kidney repair and regeneration.

TGF-β1 expression was characterized using immunohistochemistry and semi-quantitative PCR. After transplantation with the induced PBMCs, TGF-β1 expression was significantly reduced. Chronic kidney disease is a long-term process of tissue damage; renal tubular epithelial cells become dysfunctional, resulting in varying degrees of renal tubular atrophy or expansion. The expanded tubules are full of protein casts. Degeneration, hypertrophy and hyperplasia of renal tubular epithelial cells, renal interstitial fibrosis, and chronic inflammation ultimately lead to more fibrosis. Therefore, three consecutive treatments with induced PBMCs were administered in this study. By observing the ultrastructure of the renal tissue via SEM, we could also assess whether the renal function was effectively improved. The reduction in TGF-β1 expression may also reduce the extent of tissue fibrosis.

## Conclusion

Using laboratory-made natural active substances as inducing agents, adult cells were converted into iMSCs. These cells were identified using the significantly elevated expression levels of the stem cell marker genes OCT4 and NANOG. In the early stages of chronic kidney injury, iMSCs can indeed ameliorate inflammation, reduce pathological parameters of blood biochemistry, decrease kidney tissue fibrosis, increase GFR, and restore renal blood flow. For renal cell repair and regeneration, iMSCs may become one of the most promising stem cell therapies, comparable with iPSCs.
